# What Is Social Connection in the Context of Human Need: An Interdisciplinary Literature Review

**DOI:** 10.3390/ijerph22030363

**Published:** 2025-03-01

**Authors:** Kyla L. Bauer, Rachel Johnson-Koenke, Meredith P. Fort

**Affiliations:** 1 Department of Health Systems, Management and Policy, Colorado School of Public Health, University of Colorado Anschutz Medical Campus, Aurora, CO 80045, USA; 2 College of Nursing, University of Colorado Anschutz Medical Campus, Aurora, CO 80045, USA

**Keywords:** social connection, loneliness, social isolation, social determinants of health, population health, social exposome, embodiment, social homeostasis

## Abstract

The U.S. Surgeon General made an impactful declaration in the 2023 advisory on America’s loneliness and social isolation epidemic that social connection, or human relationships, is a human need equivalent to water, food, and shelter. After witnessing the impact of social isolation measures during the COVID-19 pandemic, there is a global urgency to better understand social connection in public health responses. However, meaningfully effective interventions for social isolation or loneliness have yet to be identified, and the consensus that social connection is an equivalent human need is unclear. To understand what social connection, oxygen, water, food, and shelter have in common regarding population health, we conducted an interdisciplinary literature review between September 2021 and October 2024, seeking to find commonalities between research literature advocating social connection as a human need critical to survival and key concepts across population health disciplines that explain how oxygen, water, food, and shelter function as human needs. We integrated the concepts of evolution, resource, environment, ecosystem, exposure science, embodiment, homeostasis, allostatic load theory, and interdisciplinary from 44 core publications to develop a unified conceptual model and definition for social connection as a human need. We believe a holistic understanding of social connection within the shared context of oxygen, water, food, and shelter can better support health researchers across a variety of disciplines to find common ground in developing evidence-based interventions within public health.

## 1. Introduction

In 2023, the U.S. Surgeon General declared a loneliness and social isolation epidemic affecting up to 40% of U.S. adults [1]. The U.S. is not alone, as dozens of countries and cities have developed strategies and/or action plans to address their own loneliness and social isolation epidemics [2]. These global concerns were potentially exacerbated by social isolation policies enacted during the COVID-19 pandemic [3]. Within the U.S. advisory, an important assertion was made that “social connection is a fundamental human need as essential to survival as food, water, and shelter” [1] (p. 9). While the advisory outlined a national plan for intervention, it also recognized how little is understood to effectively guide public health in addressing nationwide epidemics of loneliness and social isolation. Interventions have primarily focused on helping individuals, such as with improving social skills or receiving clinical therapy [4,5,6]. While some interventions have demonstrated effectiveness, like cognitive behavioral therapy, the small or moderate effect sizes have not yet shown full recovery into a healthy state of social connection [4,5,6]. Advisory plans like the U.S. often discussed larger concerns impacting social connection in at-risk populations, such as geographic or economic barriers, without rigorous evidence to understand these connections and largely still framing loneliness and social isolation as illnesses that require treatment rather than forms of unmet human need [1,2].

Trying to understand a newly acknowledged human need highlights that there is no existing framework in public health that cohesively explains how needs are being defined or standards for how they are addressed. ‘Human need’ is an informal phrase often used in health research that can describe anything significant to human well-being for which there is no established agreement among disciplines. Philosophers have shaped the conversation around human needs in public life, trying to distinguish moral and political obligations based on different ways to classify a need [7]. Maslow’s Hierarchy of Needs is popularly cited as defining human basic need, but it is shaped by psychology with a focus on how human motivation drives behavior that can be unrelated to population health [8]. The Agenda for Sustainable Development has created a list of what are considered basic needs to tackle global poverty using an economic lens [9]. These needs are important in modern society, but not all are required for human survival in the way that oxygen, water, food, and shelter are understood to be critical to life.

On the other hand, there is well-established knowledge in biology about why oxygen, water, food, and shelter are often considered human physiological needs because of their impact on survival. The Krebs cycle, taught as early as secondary school, explains how human metabolism relies on the presence of oxygen, water, and food to function [10]. As extreme heat events occur with climate change, innovative updates to homes and state-wide shelters have been supported by government health agencies, as shelter is known to be vital to regulating body temperatures [11].

In this way, public health interventions to address issues with physiological needs are often joint efforts with other industries or sectors, such that the scale and collection of interventions to provide a whole community with access to a single physiological need can be overlooked. A U.S. history of public health often starts with a story about the physician John Snow, who uncovered contamination in drinking water linked to cholera [12]. Efforts to ensure drinking water is safe for communities is a public health responsibility, working jointly with public water utility systems that created the pumps, wells, and infrastructure to have a water supply in the first place [13]. There is no recognized industry or sector whose primary responsibility is to provide social connection for an entire community with whom public health could work closely to address a social isolation or loneliness epidemic. Asking public health to be solely responsible for a community’s social connection could be equivalent to asking for the invention of a public water utility system, a scale and specialization for which public health agencies are not developed without identifying key partners.

The scale of interventions for human needs to improve population health requires more consideration in social connection research. Environmental biology disciplines, such as conservation biology, work together with multiple partners from different disciplines, typically at a systems scale. Water, food, and shelter are critical resources in the environment that are most effectively managed at the level of an ecosystem to support the survival of species [14,15]. Similar principles are applied to provide these resource needs for people, such as the development of a global system of agriculture to help stabilize food production regardless of environmental changes [16,17]. While efforts have been made to develop a systems approach to social connection that spans multiple disciplines, the scale and focus continue to emphasize individual or small community changes [1,18,19]. What seems to be missing is environmental and population health knowledge about needs that are critical to life, knowledge that is designed to capture and address the large-scale systems required to impact population health outcomes.

On further integrating an environmental and population health perspective with existing reviews on social connection, it can be challenging to find rigorous methods capable of doing so. Interdisciplinary and transdisciplinary literature review methods use flexibility in language to integrate shared knowledge across diverse disciplines [20,21]. These methods recognize that accuracy in scientific knowledge is not only developed by the depth of details within a single discipline but that breadth of knowledge spanning more than one discipline is equally important to understand the wholeness of a topic [20,21]. This is especially true for real-world, complex population health issues in which multiple disciplines may have overlapping and conflicting viewpoints that need to be integrated into an evidence-based strategy [22]. Interdisciplinary and transdisciplinary methods are meant to reduce the complexity of integrating knowledge by identifying and creating key points of common ground [20]. The knowledge informing these reviews is evidence-based, but the results are solely a communication tool. The main goal of an interdisciplinary literature review is to ask, “Are we talking about the same thing?” to ensure effective communication about a whole concept, like social connection, to improve collaboration across diverse disciplines and backgrounds.

An interdisciplinary literature review was conducted to further integrate environmental and population health knowledge about oxygen, water, food, shelter, and social connection that can inform public health understanding of what social connection means as a human need. In finding common ground among this group of needs, the aim of our review is to have a communication tool about the concept of social connection and how it informs public health interventions for a social isolation and loneliness epidemic. This review supports collaboration among diverse population health disciplines that may be vital to addressing an epidemic scale of unmet need for social connection in our communities.

## 2. Materials and Methods

Interdisciplinary and transdisciplinary literature reviews are conducted to better understand a complex topic with the goal of integrating knowledge across more than one discipline [20,21,23]. These reviews are not conducted to determine evidence, as their purpose is to identify common ground about a single topic that is being examined in different disciplines. They conceptually integrate shared knowledge into a unified understanding. The aim of this review is to define the concept of ‘social connection’ solely as it relates to population health and survival, limiting its definition within the shared context of oxygen, water, food, and shelter. As these human needs can be captured within many different terms across disciplines, such as ‘resources’ in environmental disciplines or within the framing of ‘social determinants of health’ in public health and social work, the literature search strategies typically employed in systematic literature review or integrative review are ineffective because they require shared terminology [14,24]. Instead, interdisciplinary literature reviews identify key literature across disciplines that are most applicable to answering a research question around a topic area. Selected knowledge across disciplines is then compared for common ground to integrate it into a cohesive concept [20,21,23]. If there are conflicting underlying assumptions or differences in terminology about the same concept across disciplines, decontextualizing the knowledge to strip it of disciplinary bias may be necessary before integration [20].

This review adapted goals outlined for conceptual integration between two or more disciplines, known as the initial steps to interdisciplinary, cross-disciplinary, or transdisciplinary research processes [20,21,23]. These goals include (1) identifying relevant disciplines, (2) identifying key literature within those disciplines to best answer a research question, (3) comparing knowledge for common ground, (4) recognizing disciplinary bias in which knowledge is produced, and (5) following an iterative process to fill in gaps in knowledge [20,21,23].

Ultimately, we followed this process to identify key concepts that capture shared knowledge in understanding social connection as a human need that impacts population health, similar to oxygen, water, food, and shelter. These key concepts are evidence-based within their respective disciplines and can be assessed for accuracy in how they are described. The process we used can also be assessed based on the scale and compatibility of integrating these concepts across the disciplines and literature we selected [23]. How these concepts are ultimately integrated into a shared understanding about a topic, like social connection, however, can only be verified as conceptual. This means that the final product of our review, the definition of social connection, solely serves as a communication aid to help researchers and practitioners understand if they are talking about the same topic to effectively work together. The definition of social connection in our results is intended to ask all those who are involved in the topic, “Is this what you mean when you use the word ‘social connection’ as a human need to improve population health?” Our final product can be assessed based on whether it makes sense of the combined knowledge and is comprehensive enough to fulfill the goals of our review [23].

### 2.1. Search Strategy

Three disciplines were selected as initial starting points in which to identify key literature to review: social neuroscience, public health, and ecology. They were selected because of their capacity to holistically define social connection within the context of human needs related to population health. Choosing three disciplines also helped to triangulate different forms of knowledge coming from disciplines that can use different research methods [20]. Social neuroscience is an interdisciplinary field that examines biological mechanisms underlying human social life to clarify the relationship between the social world and human biology [25]. Public health is an interdisciplinary field focused on improving population health within local communities through health promotion and prevention practices, which can collectively include air, water, food, shelter, and social considerations within theory about social determinants of health [12,26]. Ecology was also selected as an initial starting point because theory from ecology has been applied to both humans and all other living beings, bridging understanding across species about social relationships. Ecology is defined as the study of interaction among living organisms and their environment, including social interactions and relationships within and between species [15].

Key literature was first selected that provided substantial knowledge to holistically understand social connection within the context of human need and population health, regardless of how human need was being defined. For example, in social neuroscience, Dr. John T. Cacioppo’s book, was initially selected because it makes the claim that social connection is a human need and outlines research describing this context. In public health, a series of articles written by Dr. Julianne Holt-Lunstad were selected because they describe the impact of loneliness and social isolation on population health outcomes as key ways that overall social connection is an unmet human need. Key concepts the authors used to explain why they believed social connection to be a human need were highlighted. Additional literature was sought after that could further explain these concepts in connection to oxygen, water, food, shelter, and social connection as they related to overall population health.

In this way, literature selection was iterative, or a repetitive process, and limited in scope. Literature had to provide substantial knowledge and further understanding of key concepts that tied together social connection, oxygen, water, food, and shelter as a group, and how they related to population health or population health interventions. This means that a key concept would only be identified if it could apply to social connection, as well as water, oxygen, food, and shelter, to understand what they have in common. Literature needed to have authoritative and current scholarship to develop adequacy in key concepts and produce valid results in an interdisciplinary literature review [20]. Selection of authoritative and current knowledge to explain key concepts was aided by consulting with PhD-level experts, who could advise on authoritative researchers or significant research articles in their area of expertise [20,21]. Key concepts were identified and defined by deep reading and re-reading literature multiple times as new pieces of literature were added to the review [20]. Textbooks were the only type of literature not always read in their entirety, and instead, the chapters and chapter headings were scanned for key concepts, which would then be read deeply.

### 2.2. Consulting Experts

While interdisciplinary and transdisciplinary literature reviews recommend defining key concepts or terms in discussion with a research team, the breadth required to holistically understand social connection within the context of human need necessitated reaching out to experts beyond our research team [21]. When literature was found from outside the discipline of expertise of the research team, PhD-level experts within the discipline were contacted via email until a response was made. If unique expertise was required, then the authors of a key piece of literature or a recognized expert on a specific topic would be contacted. If broad expertise was required, such as understanding the concept of homeostasis in biology, then experts that could be conveniently reached, such as faculty who teach at local universities, were contacted. Experts were consulted to improve clarification of a key concept, to obtain their professional opinions on how it may be applied to social connection, and to recommend any further literature that should be included. Consultation is defined as providing feedback but not providing support in the design or implementation of this review [27].

The lead author continuously summarized findings of shared concepts among oxygen, water, food, shelter, and social connection as they related to population health in a working paper. Four experts offered additional time to review the working paper from September 2021 to December 2024. They are experts in multidisciplinary research in ecology, public health, social work, and systems science. These experts corrected and guided understanding about key concepts in their areas of expertise and suggested further literature to review as needed. These experts participated in at least four rounds of a review. Experts helped to correct how each key concept was being discussed based on their shared definitions (Table 1) and the accuracy of information used to construct the definition of social connection (Section 3.1, Section 3.2 and Section 3.3). Feedback was also provided for the final results of conceptual integration, but only to ensure that key concepts were not distorted from their original meaning.

### 2.3. Conceptual Integration

Upon reviewing and summarizing key literature, knowledge was compared for common ground [20]. We define common ground as consistent ideas, meaning key concepts that were discussed across multiple disciplines and multiple pieces of literature, to better understand health researchers’ claims that social connection belongs in the same group as oxygen, water, food, and shelter as it relates to population health. Key concepts we identified often represented well-established understanding in biology that was similarly discussed across disciplines in our review. Instead of needing to integrate each key concept as a research team due to conceptual differences [21], concepts shared across disciplines in our review were often already compatible and integrated (Table 1). For example, a standard definition for homeostasis was used in our review because it is recognized as the theory that the discipline of physiology is organized around [43]. Homeostasis has already been integrated across biology and social science disciplines through allostatic load theory to explain how it applies to oxygen, water, food, shelter, and social connection [46].

**Figure 1:**
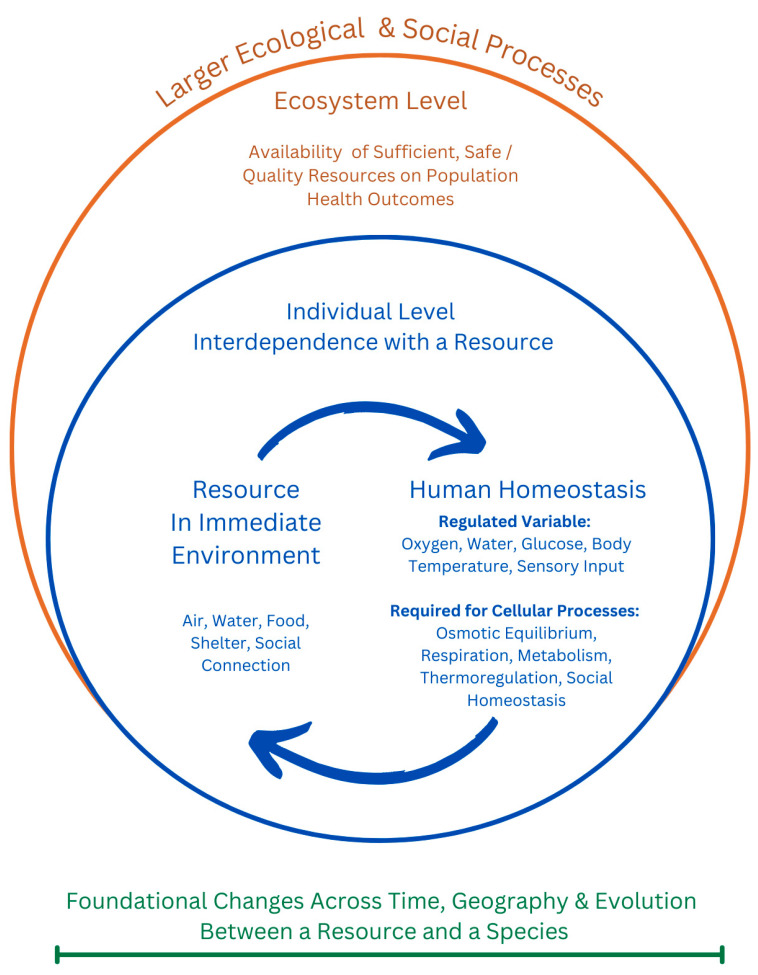
Oxygen, water, food, shelter, and social connection are resources in the environment that can be necessary for survival across species and, therefore, are important forces acting in natural selection. Green reflects the historical context of how the bi-directional relationship between a resource and a species’ survival develops. Blue is specific to people, reflecting the immediate and interdependent relationships and interactions between oxygen, water, food, shelter, social connection, and the survival and health of an individual. Orange reflects that issues with resources impacting population health can be systemic problems across an entire ecosystem. These issues can be addressed by identifying the larger ecological or social processes that may require intervention. Key concepts are further defined in Table 1.

The interdisciplinary literature review was stopped once a unified conceptual model could be produced that met our goal to holistically understand the shared context of oxygen, water, food, shelter, and social connection to inform what social connection means as a human need (Figure 1). The model visually summarized common ground in key concepts as they related to population health and theory supporting public health interventions. These concepts did not require integration because they are often already discussed together across disciplines included in our review. What is new about our conceptual model is that it makes explicit how social connection is already being evaluated and addressed across disciplines within this context. For example, homeostasis was included in the unified conceptual model to describe a primary causal pathway through which oxygen, water, food, shelter, and social connection can directly impact population health outcomes (Figure 1).

This conceptual model served as context to construct a holistic description of social connection. This means we tied together information from the literature in our review that described social connection. The goal of our definition of social connection was to have a comprehensive understanding of what social connection means in the context of a human need that impacts population health (Box 1). We further described where this information came from in Section 3.1, Section 3.2 and Section 3.3.

Box 1 **Conceptual integration of social connection.**

Social connection is an outer boundary that encompasses all direct social interactions that make up a human society. Societies may be restricted by physical exposure via location and communication, outlining an ecosystem of interdependent social interactions within and between social groups. Exposure to social interactions that can directly impact human health is the function, quality, and structure of an interaction between two or more people in a specific place. This means that social connection is a social network of contact among individuals as a group, it is the quality of interactions, and it is the role interactions can serve, such as providing informational, financial, or emotional support. Within these interactions there can be values, beliefs, and stories that can be embodied and shared across a society that inform how people interact. The social connection that makes up a human society as a whole social group is a resource critical to human survival that poses health risks across time when excluded from it, when it is of poor quality, or when it is unsafe. Outside the boundary of social connection are broader societal systems and institutions, such as an economy, policies, and cultures, that structure the social organization of a society. They may serve as a collection of interventions that help stabilize social connection for large populations. These societal structures can be misaligned with the need for social connection, as described by the social determinants of health.

## 3. Results

This interdisciplinary literature review was conducted between September 2021 and December 2024—it took over three years to conduct the review. We identified 44 core pieces of literature to include (see Supplementary Materials). Within this literature, we identified nine key concepts as having common ground to understand oxygen, water, food, shelter, and social connection as a group of needs related to population health. These nine concepts included evolution, resource, environment, ecosystem, exposure science, embodiment, homeostasis, allostatic load theory, and interdisciplinary (Table 1). There were no date restrictions on when the literature in our review was published, only that the key concepts identified in the literature included up-to-date information. Key literature predominantly included textbooks, books, government health agency reports or websites, or a collection of research articles by authoritative experts in their disciplines. Longer pieces of literature were often reviewed because they provided a holistic understanding that could not be achieved through piecemeal research articles. Literature came primarily from the disciplines of social neuroscience, public health, ecology, social epidemiology, conservation biology, and environmental and human physiology (see Supplementary Materials). Included disciplines were based in biology or had already integrated biology with the social sciences, such that conceptual integration for this literature review was often not required.

A total of 30 expert consultants were contacted, and 21 responded, including the 2 co-authors of this review. Those who responded were experts in various biology disciplines, population and human health, social sciences, and the humanities (Table 2). Only 11 recommended additional literature to include. Many expert consultants felt comfortable with labeling social connection as a human need to emphasize its importance but had not heard of the idea that it was equivalent to physiological needs. Experts helped to clarify the accuracy of key concepts included in our review (Table 1). The four experts who provided additional review also reviewed the accuracy of the information included in Section 3.1, Section 3.2 and Section 3.3, which further explained the knowledge we used to inform our definition of social connection. Experts did not validate the evidence for our final conceptual model (Figure 1) or our final definition of social connection (Box 1) because conceptual integration emphasizes common ground, not evidence, that can change how disciplines interpret a topic. Conceptual integration is not being used to determine evidence for a topic. These final products are instead intended to improve communication about how this topic is being addressed across disciplines in similar ways to better support collaboration.

When discussing the goal of the review to integrate understanding about the needs of water, oxygen, food, shelter, and social connection, however, many experts expressed discomfort and often suggested getting additional input from social science and humanities experts. One social science expert was opposed to discussing ideas from biology in connection to sociology. An expert in bioethics and humanities was consulted, who provided feedback on the tension being uncovered in our review and suggested additional literature. We added more literature to our review from philosophy and public health ethics about human needs (see Supplementary Materials) [7,47].

Based on the nine concepts listed in Table 1, the following is a brief explanation of common ground found among oxygen, water, food, shelter, and social connection:

These human needs reflect a relationship between a resource in the environment and humans as a species, relationships that have played a significant role in our evolution and are encoded in our genes. The expected functioning of homeostasis in humans remains dependent upon physical exposure to these resources (Figure 1).

The unified conceptual model was used to frame a definition of social connection (Box 1). It was constructed from common ground in shared conceptual knowledge across the literature included in our review. The following subsections explain information that was used to construct our definition of social connection. We found three key ways that health researchers define the concept of social connection across the disciplines included in our review. Each section details a key way to conceptually understand social connection.

### 3.1. How the Evolution of Social Species Is Being Used to Define Social Connection

All animals could be described as social (personal communication, A. Kamath, Animal Behavior expert, 11 November 2022). Animals are characterized by their ability to move and have a degree of autonomy in choosing how to move based on their interactions with other species and natural resources [28]. Social interactions include interactions between species that ecology can categorize by negative, positive, or neutral qualities [15]. For example, parasitic interactions describe when the health and survival of one species depends on the reduced health of another. Mutualism describes when the health and survival of two different species benefits from their interactions. Social relations and sociality, though, more narrowly focus on interactions within a species that impact survival, such as sex, and when species become dependent on group living and cooperation [15].

There has been an effort to formally define social species through the development of sociobiology by Dr. E. O. Wilson, who studied the evolutionary biology of social insects [45]. He attempted to systematically define the shared biology and behaviors of social species, including humans, to better understand the evolutionary reasons for dependence on group living as a critical resource for survival. His main definition of a social species was the ability to form a society that organized cooperative interactions within a single species through communication [45]. Any expansion on this definition was challenging because of the variety in social behaviors between different families and genera, as well as complex versus simple animal species. Continued efforts to organize and understand social behaviors across social species more often cite Tinbergen’s Framework, which recognizes that social species exist on a spectrum rather than as a binary category [15,48]. The framework highlights that social behaviors can be produced through mechanisms, have a role in a species’ developmental stages and evolutionary history, and can impact survival. Ethical concerns can often be raised in this area of work about integrating human social life into traditional biology interpretations of animal social behaviors [49,50]. Racist and homophobic ideologies, for example, have been promoted in the past by specific biologists who studied social behaviors, and these concerns remain a key challenge in integrating knowledge [45,49,50].

Health researchers have theorized that since human beings are a “social species” (p. 438), social connection is likely to be a critical part of human physical health [18]. The U.S. Surgeon General has defined the human need for social connection as the “size and diversity of one’s social network and roles, the functions these relationships serve, and their positive or negative qualities” [1] (p. 7). This definition of social connection could be interpreted as solely one’s network of relationships with friends and family. However, the advisory further explains that it is taking a broad approach to include any interaction or relationships with an individual as part of a community and society. The evolutionary theory of social connection is highlighted to explain the critical nature across human history of becoming isolated from a whole social society, which could result in death [1,25].

In this context, there is a shared understanding that social connection for humans is occurring at the level of human society and an individual’s dependence on the collective social interactions within it. However, it is challenging to understand the defined boundaries of a society in the context of modernity today. Dr. Wilson defined the outer boundaries of society for a social species based on rates of communication and steep drop-offs in communication between populations [45]. In public health, society is often defined by the social–ecological model that describes the organization of social groups as an ecosystem that is known to impact human developmental and health outcomes [37,39]. The social ecological model captures the whole and direct social context in which people live, work, and play [37,39]. The U.S. Centers for Disease Control and Prevention has updated the understanding of society in the model to include cultural and social norms, as well as policy that creates socio-economic conditions [37]. This creates two possible boundaries—one at the boundary of a human society in which there are direct social interactions and one at the boundary of a society that can be as large as a nation-state, in which federal policies and national norms can be enacted.

### 3.2. How Direct Social Interactions Causing Population Health Outcomes Is Being Used to Define Social Connection

Social epidemiology is a subdiscipline “concerned with the way that social structures, institutions, and relationships influence health” [51] (p. 2). Dr. Emile Durkheim, a sociologist, is cited as an early influence on developing social epidemiology. He studied the importance of social environments on suicide outcomes [51,52,53]. He considered the composition of social groups and communities to prevent suicide instead of solely focusing on individual behavior [51]. Social epidemiology, community psychology, and social work have continued to focus on the influence of larger social groups, such as families and neighborhoods, to understand core social circumstances contributing to a wide range of diseases [51]. They have found that a variety of factors that describe human social life, like socioeconomic status, can have significant impacts on population health outcomes [51].

However, understanding the health impacts of social connection has selected for social factors that only describe direct interactions within social groups [1]. Predominant among them are social isolation and loneliness because they independently contribute to increases in all-cause mortality risk [54,55,56,57]. Social isolation and loneliness represent two distinct ways in which social connection as a whole can be lacking for individuals. Social isolation describes an objective lack in total quantity of human relationships and low contact with others [58]. Loneliness is often defined as a discrepancy in overall relationship qualities that are undesired and the feeling of being alone, even if someone is not socially isolated [55,59,60,61]. In reverse, social factors that collectively help describe the presence of social connection refer to the structure, function, and positive qualities of social interactions [1]. Factors like social support, trust, or social networks of interconnected relationships among groups can describe ways of having social connection [1].

The reason for focusing only on direct social interactions when describing social connection may have to do with identifying the causal mechanism for how social connection impacts health outcomes. While a bio-psycho-behavioral model describing three pathways for social connection to impact health was proposed by the U.S. Surgeon General, these pathways can be interdependent and difficult to distinguish [1,51]. Instead, the primary causal pathway consistently mentioned across disciplines and literature in our review is that social connection impacts population health outcomes through homeostasis and/or allostasis [25,30,35,39,45,46,51].

Homeostasis is defined as a “self-regulating process by which an organism can maintain internal stability while adjusting to changing external conditions” (p. 2) [43]. It encompasses a number of feedback loops that help to regulate essential needs, such as the feeling of hunger and feeling full as a feedback loop to regulate an essential amount of food for human survival [25,62]. The disruption of its feedback loops can directly develop into disease and/or mortality risk [43]. The human brain is involved in identifying internal and environmental changes that stress the ability to maintain homeostasis, responding through mediators that expand the body’s capacity to withstand change, called allostasis [44,63,64]. This type of stress is directly related to the deprivation of resources in the environment, such as oxygen, water, food, and shelter, that are used to meet tightly regulated physiological needs essential to human life [44].

Historically, social homeostasis was described as an external feedback mechanism found in social species who could use collective behaviors in groups to regulate the internal physiology of the whole social group [30,45]. In humans, it aligns with family systems theory, which describes the different roles and functions of each family member as a way to regulate overall stress levels of the whole family [65]. Disease symptoms of an individual could worsen or be alleviated by the physical presence of their family [65]. Dependence on social groups to alleviate stress has been described in households, neighborhoods, workplaces, and schools, which demonstrates stress dependency on beneficial, stable, and present social interactions compared to harmful or violent, chaotic, or neglectful social interactions [46]. Social homeostasis is being updated to examine internal neural feedback mechanisms that may depend on stimulus from having physical proximity to social resources that help the human brain maintain a resting state [30,31,66]. The human brain seems to expect an immediate social environment that can contribute to distress when it is not present, such as being in social isolation. This means the human brain is potentially treating social resources similarly to glucose or oxygen. It is believed a neural feedback mechanism could exist across animals who live in social groups because social groups and behaviors can benefit risk-sharing, labor-sharing, and resource-sharing [30].

A physical exposure to social resources, and then their biological embodiment that impacts health outcomes, is often described through sensory input from direct social interactions. The social exposome model hypothesizes that the primary pathway through which the social world is embodied in humans is through social interactions between two or more people occurring in a specific place and their dynamics [39]. Exposure to the sound of language impacts a child’s neurocognitive development [39]. Premature babies in neonatal care units fail to live when they lack touch, even though they have other basic needs met [66]. Severe social deprivation of institutionalized children in Romania resulted in reduced brain activity, which was only improved if children were placed into a community through foster care at earlier ages [67]. Significant changes in brain structure have been found in children to adapt to abusive or neglectful household environments, which may depend on the touch and verbal nature of maltreatment [68]. Even a social group quality as abstract as gender norms or racism can be learned and internalized through physical exposure to social interactions in which a culture’s norms are enforced [39,42,69].

Information about the causal pathway from social connection to human health is not being mentioned to provide evidence for a specific pathway. Instead, our goal is to explain consistent concepts found across disciplines that are being used to define social connection. Literature in our review limits the concept of social connection to encompass any characteristic that describes direct social interactions because it is thought that direct exposure to a social environment is necessary to have it impact human health. This would be similar to the concept of food encompassing any characteristics about animals or plants that people consume to support nutrition needs.

### 3.3. How Population Health Interventions for Critical Resources Are Being Used to Define Social Connection

Social determinants of health (SDOH) have become a central focus of public health interventions that consider larger societal structures, such as an economy, policies, and cultures, as interconnected systems that shape social relationships within a human society [70]. These systems can contribute to objective differences in social and environmental living conditions that create objective differences in population health [33,37,71,72]. This means that systems that organize people within human societies can determine the immediate environments in which people live, work, and play that directly impact human health through exposure. A common example describes social economic systems in which income is earned through jobs, where low-income workers can experience a clustering of SDOH, like being more likely to be exposed to unsafe work environments, living in unsafe housing, and having limited purchase and time for essential needs and social participation in their communities [35,73]. Allostatic load theory is often cited in the SDOH framework as a biological mechanism to explain how both social and material living conditions in an immediate environment can directly result in poor population health outcomes [46].

**Figure 2:**
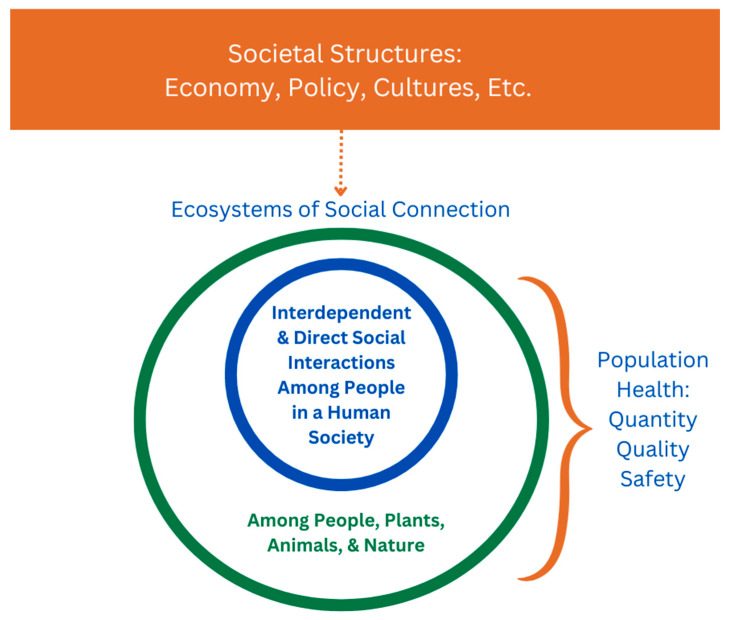
A re-interpretation of the social determinants of health that centers solely around social connection. In orange: the existing and interconnecting systems that help stabilize social connection in human societies. In blue: a human society composed of social interactions among people that are species-specific to humans. In green: a human society composed of social interactions between people and any other living species within the same ecosystem, which can provide social connection regardless of species, known as being function-specific. Aligning societal structures with the quantity, quality, and safety of social connection in society would be key considerations for intervention in public health.

While SDOH are often interpreted negatively by highlighting how differences in population health outcomes are unfairly produced, it may be overlooked that this framework is describing an ecosystem centered around human relationships. The very existence of systems that organize a human society, like an economy, a government that enacts policies, and cultures, could be how social connection needs are formally supported for populations as large as nation-states (Figure 2). This is an existing gap in our literature review; to clarify if societal systems can determine either positive or negative population health outcomes based on how people are socially organized in a society, do they hold primary responsibility for social connection?

To think through this question, it may help to understand what the SDOH framework has in common with an ecosystem management approach used in conservation biology [14,15]. Due to having an environmental focus on preventing the untimely extinction of a variety of species, conservation biology is concerned with providing resources that are critical to fulfilling the physiological needs for a species’ survival [14]. Ecosystem management can be used to stabilize the quantity, quality, and safety of a resource to improve access [14]. The provision of physiological needs for individuals or even specific populations has not been effective at improving a species’ population health, requiring consideration of the entire ecosystem as it applies to the whole population living there. Systems science helps identify key elements to focus on in the ecosystem in order to facilitate the greatest overall improvement for a given species [15]. These elements are multidisciplinary, involving biological and physical sciences, as well as social sciences and policy [14].

The ecosystem-level management of the quantity, quality, and safety of resources is also a common consideration in public health [34]. Agriculture is a global system of interventions that support the world’s population by ensuring sufficient calories from foods that are safe and nutritious [74,75]. The role of public health has worked to align agriculture with population health priorities in nutrition [75]. Air and water quality across large geographic regions are regulated by government environmental agencies to minimize pollution for the safety of their consumption and to manage how they are shared among diverse interests within a region [76,77]. While the U.S. Surgeon General is urging that the quantity and quality of social connection be addressed in public health [1], the safety of social connection is already being addressed through violence prevention policies and programs [37].

SDOH frameworks recognize a misalignment between societal structures, like economies, policies, and culture, with human well-being [24,33]. These frameworks have historically described social connection concerns at a societal level, like the impact of social exclusion of individuals or communities from a society by societal structures, a social exclusion that can be intertwined with the ability to access overall needs [35]. An update has been suggested that re-emphasizes the central role of social connection for the whole framework [19]. Shared practices in conservation biology and public health suggest thinking even more holistically about societal structures, that the very reason they came into existence is to support social connection in larger populations, and how they may need to be examined for their ability to sustain the quantity, quality, and safety of social relationships that make up a human society.

A final consideration in SDOH is that human relationships are not exclusive to humans. Systematic literature reviews of social isolation and loneliness interventions for older populations have focused on improving meaningful relationships through peer support or social groups, as well as animal or pet therapy programs, gardening with plants, hobbies with art and music, and exercise [6,78]. During social distancing policies through the COVID-19 pandemic, there was higher visitation to green spaces and parks, which are considered forms of social connection with nature [79,80]. Conservation biology would deem this a functional approach to social connection in which a variety of species could fulfill a similar role in an ecosystem [14]. For example, if social connection is experienced through sensory input, the touch of petting a dog and the sound of its bark or the smell of flowers while gardening may explain why these relationships can help fulfill social connection needs in people (Figure 1). Understanding the total quantity, quality, and safety of social connection in the ecosystems in which people live, including relationships with nature and other living things, may better define social connection.

## 4. Discussion

The goal of this interdisciplinary literature review was to improve communication across health disciplines in developing a shared understanding about social connection as a human need that impacts population health. To be clear, when the U.S. Surgeon General is calling for the equal prioritization of social connection, water, food, and shelter in public health, it is because health researchers are examining social connection as a physiological human need. Homeostasis is a core concept to understand how physiological needs are linked to population health, with social homeostasis specifically applying to social connection. Social homeostasis, which describes the physiological dependence of social species on social groups, was first recognized in 1956 [45]. Advances in neuroscience have begun to test social homeostasis as an internal physiological feedback system in humans [30]. Finding common ground between social connection and the physiological needs for oxygen, water, food, and shelter was not an issue in our review. Instead, the challenge in our review was understanding what was so different about social connection. Why is there no consensus on whether or not it belongs in this group as a physiological need?

While consulting experts in our review often expressed agreement with the U.S. Surgeon General’s Advisory that acknowledging social connection as critical to human health is long overdue [1], none had heard the idea that social connection should be prioritized equivalently to physiological needs. In communication with experts, there was a noticeable tension between experts in population health who work on environmental issues that impact the health of our air, water, food, etc., and social scientists who work on social and relationship health, about combining their knowledge. Our review suggests that a primary obstacle in better understanding social connection as a human need is ethical concerns about integrating knowledge on human social life with how biology frames and treats physiological needs for population health.

There has been a long-standing ethical division between biology and the social sciences in how to study and apply knowledge regarding the survival of social species to human social life [49,81]. Historical concerns about how evolutionary biology has been interpreted to support human harm, like eugenics, has contributed to some social sciences excluding any biology knowledge from their work [81]. This can complicate understanding the biology of social connection as a human need. Interdisciplinary efforts have been necessary for health researchers to bridge the divide. For example, a collaboration between the neuroscientist, Dr. McEwen, and his brother, the sociologist Dr. McEwen, was necessary to overcome disciplinary distrust and explain the role of social inequality in contributing to population differences in physiological stress from unmet need [46]. Similarly, subdisciplines like social epidemiology have been created to integrate social science understanding with biology to address the impact of social connection on population health [51].

The importance of considering whether or not social connection is a physiological need is that it impacts guidance on how to develop effective interventions. Theory already exists about potential interventions that have been successful in addressing physiological needs in the environment to improve population health outcomes, but there is tension on how or if it can be applied to social connection. For example, the U.S. Centers for Disease Control and Prevention has prioritized improving how many adolescents have an adult to talk to and reducing depression in family caregivers for those with disabilities as specific targets to improve social connection [82]. These goals can reflect a singular and linear approach to social connection at a population health level, but they do not meet the systems requirements to effectively address a critical resource for a physiological need in a whole population. Instead, the conceptual understanding of physiological need from our review suggests that larger social processes across a society living together would need to be identified for intervention if there are issues with the overall quantity, quality, and/or safety of social connection. A systems intervention would focus on improving larger social processes in a social environment that cannot be disentangled from each other, rather than treating lonely or socially isolated individuals.

For example, loneliness research has identified that populations going through significant transitions in their social environments due to life changes are experiencing critical times for increased loneliness [83,84,85]. It is a shared, larger social process for loneliness when graduating young adults are trying to join the workforce, Veterans are transitioning from military to civilian life, or when certain ages commonly experience the loss of significant relationships. Another example is the larger social process of deviating from cultural norms that seems to increase the risk of loneliness, such as being a single adult when expected by society to be married [86]. Larger social processes may identify ways that a significant minority of a population can end up becoming stuck or overwhelmed in severe or chronic loneliness, regardless of individual factors. It may illuminate why national policies to address loneliness and social isolation epidemics are finding that at-risk populations seem to also struggle with social determinants of health, like being unemployed or unable to access transportation [2]. To improve guidance for public health, instead of identifying individual risk factors that can appear to be separate considerations for different populations, it may help to examine the larger social processes beneath them, which explain how diverse populations within a society are struggling with social connection for similar reasons.

In our review, we reinterpreted the social determinants of health framework as a way to think about the central role of social connection in our societies and which larger social systems may require intervention to ensure sufficient quantity, quality, and safety. It is just one example, though, of a framework that can guide systems interventions for addressing social connection as a human need. Another example found in our review is the social exposome model. It is a systems framework to assess physical exposure to social connection in a society [39]. It is able to connect larger social processes with population health outcomes. Within systems science, there are several frameworks that could help public health experts identify ways to restructure social systems, like policymaking, to improve social connection at the scale of local and national epidemics [87].

In summary, the value of defining social connection as a human need based on shared concepts across disciplines is to improve collective standards for how to approach the loneliness and social isolation epidemic. The definition from this review should not be translated as the ‘right’ way to understand social connection but to aid discussion about why community interventions will be effective and for how many people. Is an intervention focused on specific kinds of relationships to improve social connection, or is it considering any kind of social connection that can be found within a society? At what level of social group is an intervention designed and why—such as changing individuals, changing neighborhoods, changing institutions, or changing societal structures—since these levels are not identical. Has a larger social process driving loneliness and social isolation across a whole community been identified for intervention? Asking these kinds of questions can expand our understanding of what effective loneliness and social isolation interventions look like. A change in policy to raise the minimum wage, if it improves the capacity of low-income workers to have a social life, could be more impactful on the loneliness and social isolation epidemic than further improvements in therapeutic or small-group treatments.

In terms of limitations, our review is not being used to determine if social connection is a human physiological need but to have a shared conceptual understanding of how social connection is being evaluated as a human need across disciplines interested in improving population health outcomes. The goal of our interdisciplinary literature review was not to determine evidence for a research topic but to identify conceptual common ground across disciplines that are engaged in the same research topic [21]. To address social connection at the scale of a national epidemic, it is a necessary step to develop a shared understanding that can build mutual goals and efforts in research and practice.

Methodological guidance for interdisciplinary and transdisciplinary literature reviews is harder to locate compared to guidance for systematic or integrative literature reviews. Methodological guidance was additionally challenging because it is often abstract, making it difficult to evaluate how to do a rigorous interdisciplinary review [23]. However, the iterative process of comparing oxygen, water, food, shelter, and social connection as human needs and consulting with many disciplinary experts resulted in the inclusion of key literature and concepts from social epidemiology, public health, ecology, conservation biology, environmental or human physiology, social neuroscience, ethics, and philosophy. We also explicitly stated in our methods section the ways that our review can be evaluated for accuracy and rigor to make clear how to assess our approach.

We found the concept of social connection as a human need to be a much bigger idea than anticipated, but its critical impact on population health deserves the attention of research. Interdisciplinary and similar forms of literature reviews may be the only kinds of research methods capable of developing a conceptual understanding of social connection because they are designed to support a “grand challenge” of knowledge integration (p. 62) [23]. However, our review was not intended to be exhaustive. It was intended to be sufficient in producing a unified conceptual model to understand the shared meaning of social connection across key disciplines within the context of oxygen, water, food, and shelter as they relate to population health. While much of the literature included in our review had already integrated social science and humanities perspectives with biology, our results may still emphasize a biology perspective on population health that requires further integration.

Due to the large scope of a human need, the goal of this review was breadth in integrating key concepts across several disciplines rather than explaining social connection in depth. This aligns with the goals of interdisciplinary integration [20,21,23]. For readers wanting greater depth, we highlight key literature and key research authors who have already provided in-depth reviews within their disciplines, as suggested in further reading in our Supplementary Materials. Regardless, our review did not examine a multicultural definition of social connection that can change across cultures. It also did not sufficiently consider the public health priority of equity in the need for social connection.

## 5. Conclusions

Placing social connection in the context of human need does not seem to impact what is already understood about human social life and the resulting consequences on population health, but it does make the biology and physicality of social connection more visible. When social connection is examined holistically as the composition of all direct social interactions that make up a society, it is easier to understand why the U.S. Surgeon General is asking for equal priority to address unmet social connection as other human physiological needs. In addition, health researchers across several disciplines were found to be applying concepts about human physiological needs to social connection. This review can be used as a communication tool to support larger interdisciplinary conversations about testing and developing interventions at the scale of loneliness and social isolation epidemics. By centering social connection within an applied framework like social determinants of health, we can think about the implications for institutions and societal arrangements that are needed to support social connection in our communities. In the way we think of basic needs like water, air, food, etc., as shared resources, public health can move beyond a clinical and individual lens to view social connection at a broader ecological level. Our review suggests to social connection practitioners to think on much larger scales about interventions and about what is needed to shift societally to have sufficient social connection in our communities.

## Figures and Tables

**Table 1:** Definitions of key concepts used to construct a unified conceptual model for oxygen, water, food, shelter, and social connection.

Key Concept	Shared Definitions Found Across Population and Human Health Disciplines, and Application to Social Connection
Evolution	Evolution is defined in biology as a change in gene frequencies within a species over time that results from interactions with the environment [15]. It can arise by several mechanisms, including natural selection from the environment. Such pressures have influenced the evolution of many traits, such as ways of accessing food sources and maintaining oxygen levels, body temperature, and water balance [15,28]. Traits of social connection for humans, such as the capacity to feel lonely, are also genetically based [1,25,29,30]. It is theorized that social connection may be associated with core neural mechanisms developed through evolution across animal species exhibiting social behaviors [30]. Social connection unique to humans developed in early human ancestors who may have been more likely to survive by living in groups because of associated benefits, such as protection or acquiring food [1,25].
Resources	Natural resources refer to any commodity or quality found in nature, such as water, materials for buildings, wildlife and plants for food, etc. [14]. Social resources describe the social ecology that the human brain seems to expect to function in, resulting in stress when it is not present [31]. Human social bonds within larger social groups have been found to be critical to human survival across human history [1,25].
Environment	In ecology (including conservation biology), the environment of an organism includes all features that affect it, often emphasizing abiotic (nonliving) elements, such as moisture levels or temperature [15]. Ecology as a field is defined as the study of the interaction among organisms and their environment. In public health, environment is similarly considered to be the external conditions that influence human health [32,33,34]. A social environment refers specifically to the interactions among people that influence human health, such as the interaction of social and economic conditions [32,35].
Ecosystem	A system describes the dynamic interactions of individual components that function as a whole, such as the interactions of a biological (living) community and the physical environment that make up a whole ecosystem [15,36]. For example, an urban area that includes the collection of biological, physical, and social elements is an ecosystem [15]. An ecosystem also considers larger processes that impact communities and their physical environment [15]. While ecology may focus on processes like energy flows in an ecosystem, public health often focuses on social processes, such as cultural influences, and economic or public policies and their impact on population health [34,35,37,38]. In public health, the combined biological, physical, and social living conditions of human well-being are described within the social determinants of health. The social–ecological model also uses ecology concepts to organize human social life as a system.
Exposure Science	Exposure science is described in public health as the study of physical contact with environmental factors, such as by swallowing, breathing, or touching them, and the effects of their exposure on human health [32]. It can also be integrated into social epidemiology and overlaps with core understanding in ecology and physiology [15,28,39]. External environmental factors considered in population health can include air, water, food, housing environments, work environments, and social interactions [32].
Embodiment	Embodiment is described in social epidemiology when humans “literally incorporate, biologically, the environment in which we live, including societal and ecological conditions” (p. 351) [40]. It corresponds with exposure to environments that people interact with using agency, resulting in variation in biological characteristics across populations due to the variation of physical, chemical, biological, or social exposures that are embodied [40,41]. Embodiment integrates core understanding from ecology and physiology, but it highlights the importance of politics and the economy as structural sources of injustice in exposures that can impact the distribution of poor health [41,42]. Exposure science has been combined with embodiment to describe how direct social interactions between people can become embodied, theorizing a direct biological pathway from social injustices, like racism, to human health [39].
Homeostasis	Homeostasis is a core part of physiology, and it is mentioned across disciplines in our review. It is “a self-regulating process by which an organism can maintain internal stability while adjusting to changing external conditions” (p. 2) [43]. Oxygen, water, glucose, and body temperature are tightly regulated through feedback mechanisms to maintain critical levels in the human body that are needed to survive [43,44]. Social homeostasis was originally described as an external maintenance of stability at the level of society of a social species that can regulate the physiology of group members as a whole [45]. It has been updated to test neural mechanisms that internally regulate social connection across all animals exhibiting social behaviors, in addition to humans [30].
Allostatic Load Theory	A biology term used across human health disciplines to further explain the impact of homeostasis on human health outcomes. When factors regulated by homeostasis fall outside necessary ranges, it is an acute stress to which the human body is capable of adapting through a temporary allostasis response [44]. Environmental conditions, such as pollution, unstable housing, economic conditions, racism, abuse, or neglect, can contribute to a chronic allostasis response that results in greater wear and tear on the body over time [26,37,44].
Interdisciplinary	The reliance on multiple disciplines to holistically understand the complex interactions taking place in an ecosystem, whether biological or societal, by integrating knowledge across disciplines [12,14,15,19,25,29,42].

**Table 2:** Disciplinary expertise of consultants (N = 21).

Discipline	Count ^1^
Conservation Biology	3
Ecology	5
Medicine	2
Public Health: Environmental Health	3
Public Health: Bioethics & Humanities	1
Public Health: Health & Behavioral Science	5
Public Health: Health Systems	4
Social Science	6
Systems Science	4

^1^ Each consultant may have more than one area of expertise.

## Data Availability

No new data were created or analyzed in this study. Data sharing is not applicable to this article.

## Supplementary Materials

The following supporting information can be downloaded at: https://www.mdpi.com/article/10.3390/ijerph22030363/s1, Table S1: Suggested further reading of key sources of literature, authors and disciplines from interdisciplinary literature review.
