# Supplementary material for: What Is Social Connection in the Context of Human Need: An Interdisciplinary Literature Review
Source: Int J Environ Res Public Health. 2025 Mar 1;22(3):363. doi: 10.3390/ijerph22030363 (PMC11941835; doi:10.3390/ijerph22030363)
Supplement: Supplementary file 1 [file ijerph-22-00363-s001.zip › ijerph-3408120-supplementary.pdf]

Supplementary Material

Suggested Further Reading of Key Sources of Literature, Authors & Disciplines From Interdisciplinary Literature Review

**Topic Areas**	**Key Literature Reference**
Loneliness & Social Isolation Epidemic	U.S. Department of Health and Human Services. *Our Epidemic of Loneliness and Isolation: The U.S. Surgeon General's Advisory on the Healing Effects of Social Connection and Community*. 2023; Available from: https://www.hhs.gov/sites/default/files/surgeon-general-social-connection-advisory.pdf [1].
Homeostasis	Modell, H., et al., *A physiologist's view of homeostasis.* Adv Physiol Educ, 2015. **39**(4): p. 259-66 [2].<br><br>Billman, G.E., *Homeostasis: The Underappreciated and Far Too Often Ignored Central Organizing Principle of Physiology.* Frontiers in Physiology, 2020. 11 [3].
Social Homeostasis	Cacioppo, J.T. and W. Patrick, *Loneliness: Human nature and the need for social connection*. 2008, New York, NY and London, England: W. W. Norton & Company. 264 [4].<br><br>Coan, J.A. and D.A. Sbarra, *Social Baseline Theory: The Social Regulation of Risk and Effort.* Curr Opin Psychol, 2015. **1**: p. 87-91 [5].<br><br>Matthews, G.A. and K.M. Tye, *Neural mechanisms of social homeostasis.* Ann N Y Acad Sci, 2019. 1457(1): p. 5-25 [6].<br><br>Fulford, D. and D.J. Holt, *Social Withdrawal, Loneliness, and Health in Schizophrenia: Psychological and Neural Mechanisms.* Schizophr Bull, 2023. 49(5): p. 1138-1149 [7].
Social-Ecological Model	Bronfenbrenner, U., *Toward an experimental ecology of human development.* Am Psychol, 1977. 32(7): p. 513-531 [8].

	U.S. Centers for Disease Control and Prevention. *The Social-Ecological Model: A Framework for Prevention*. April 9, 2024; Available from: https://www.cdc.gov/violenceprevention/about/social-ecologicalmodel.html [9].
Social Exposome	Gudi-Mindermann, H., et al., *Integrating the social environment with an equity perspective into the exposome paradigm: A new conceptual framework of the Social Exposome.* Environ Res, 2023. 233: p. 116485 [10].<br><br>The National Institute of Environmental Health Sciences. *Exposure Science*. October 2, 2024; Available from: https://www.niehs.nih.gov/health/topics/science/exposure/index.cfm [11].
Social Determinants of Health	Raphael, D., *Social Determinants of Health: Canadian Perspective*. 3rd ed. 2015, Toronto, Ontario: Canadian Scholars Press Inc [12].<br><br>Raphael, D. and T. Bryant, *Emerging Themes in Social Determinants of Health Theory and Research.* Int J Health Serv, 2022. **52**(4): p. 428-432 [13].<br><br>Shultz, J.M., et al., *Public Health*. 2 ed. An Introduction to the Science and Practice of Population Health, 2023. New York: Springer Publishing Company [14].<br><br>Muntaner, C. and J. Benach, *Why social (political, economic, cultural, ecological) determinants of health? Part 1: Background of a contested construct.* International Journal of Social Determinants of Health and Health Services, 2023. **53**(2): p. 117-121 [15].<br><br>Centers for Disease Control and Prevention. *Social Determinants of Health*. January 17, 2024; Available from: https://www.cdc.gov/about/priorities/why-is-addressing-sdoh-important.html [16].<br><br>World Health Organization. *Social Determinants of Health*. 2024; Available from: https://www.who.int/health-topics/social-determinants-of-health#tab=tab_1 [17].
Philosophy of Human Need	*A Philosophy of Need*, ed. S. Reader and G. Brock. 2024, Cambridge, UK: Cambridge University Press [18].

Public Health Ethics of Social Determinants of Health	Goldberg, D.S., *Public Health Ethics and the Social Determinants of Health*. 1st ed. 2017, Aurora, CO: SpringerBriefs in Public Health [19].

Key Authors	Career Research	Key Literature Reference
J. Holt-Lunstad	Disease & Mortality Risk of Loneliness & Social Isolation	Holt-Lunstad, J., T.B. Smith, and J.B. Layton, *Social relationships and mortality risk: a meta-analytic review.* PLoS Med, 2010. 7(7): p. e1000316 [20].<br><br>Holt-Lunstad, J., et al., *Loneliness and social isolation as risk factors for mortality: a meta-analytic review.* Perspect Psychol Sci, 2015. 10(2): p. 227-37 [21].<br><br>Holt-Lunstad, J., *Why Social Relationships Are Important for Physical Health: A Systems Approach to Understanding and Modifying Risk and Protection.* Annu Rev Psychol, 2018. 69: p. 437-458 [22].<br><br>Holt-Lunstad, J., *Social Connection as a Public Health Issue: The Evidence and a Systemic Framework for Prioritizing the "Social" in Social Determinants of Health.* Annu Rev Public Health, 2022. 43: p. 193-213 [23].
B.S. McEwen & C.A. McEwen	Homeostasis & Allostatic Load Theory	McEwen, B.S., *Stress, adaptation, and disease. Allostasis and allostatic load.* Ann N Y Acad Sci, 1998. 840: p. 33-44 [24].<br><br>McEwen, B.S., *Stressed or stressed out: what is the difference?* J Psychiatry Neurosci, 2005. 30(5): p. 315-8 [25].<br><br>McEwen, B.S. and P.J. Gianaros, *Stress- and allostasis-induced brain plasticity.* Annu Rev Med, 2011. 62: p. 431-45 [26].<br><br>McEwen, C.A., *Connecting the biology of stress, allostatic load and epigenetics to social structures and processes.* Neurobiol Stress, 2022. 17: p. 100426 [27].
J.T. Cacioppo & L.C. Hawkley	Loneliness in Social Neuroscience	Hawkley, L.C., M.W. Browne, and J.T. Cacioppo, *How Can I Connect With Thee?: Let Me Count the Ways.* Psychological Science, 2005. 16(10): p. 798-804 [28].

		Cacioppo, J.T. and W. Patrick, *Loneliness: Human nature and the need for social connection*. 2008, New York, NY and London, England: W. W. Norton & Company. 264 [4].<br><br>Hawkley, L.C. and J.T. Cacioppo, *Loneliness matters: a theoretical and empirical review of consequences and mechanisms.* Ann Behav Med, 2010. 40(2): p. 218-27 [29].<br><br>Cacioppo, S., et al., *Loneliness: clinical import and interventions.* Perspect Psychol Sci, 2015. 10(2): p. 238-49 [30].<br><br>Cacioppo, J.T. and S. Cacioppo, *The growing problem of loneliness.* Lancet, 2018. 391(10119): p. 426 [31].
N. Krieger	Embodiment	Krieger, N., *Embodiment: a conceptual glossary for epidemiology.* J Epidemiol Community Health, 2005. 59(5): p. 350-5 [32].<br><br>Krieger, N., *Discrimination and health inequities.* Int J Health Serv, 2014. 44(4): p. 643-710 [33].<br><br>Krieger, N., *Living and Dying at the Crossroads: Racism, Embodiment, and Why Theory Is Essential for a Public Health of Consequence.* Am J Public Health, 2016. 106(5): p. 832-3 [34].<br><br>Krieger, N., *Theorizing epidemiology, the stories bodies tell, and embodied truths: a status update on contending 21(st) c CE epidemiological theories of disease distribution.* Int J Soc Determinants Health Health Serv, 2024. 54(4): p. 331-342 [35].

**Key Disciplines & Sub-Disciplines**	**Literature Type**	**Reference**
Conservation Biology	Sher, A.A., *Introduction to Conservation Biology*. 3rd ed. 2022: Oxford University Press [36].	
Ecology	Sher, A.A., Molles Jr., MC, *Ecology: Concepts and Applications*. 9th ed. 2022, New York, NY: McGraw Hill. 547 [37].	
Environmental / Eco Physiology of Animals	Willmer, P., G. Stone, and I. Johnston, *Environmental physiology of animals*. 2009: John Wiley & Sons [38].	
Public Health	Shultz, J.M., et al., *Public Health*. 2 ed. An Introduction to the Science and Practice of Population Health, 2023. New York: Springer Publishing Company [14].	
Social Epidemiology	Book	*Social Epidemiology*. 2nd ed, ed. L. Berkman, Kawachi, I, Maria Glymour, M. 2014, New York, NY: Oxford University Press [39].
Social Neuroscience	Books	Cacioppo, J.T. and W. Patrick, *Loneliness: Human nature and the need for social connection*. 2008, New York, NY and London, England: W. W. Norton & Company. 264 [4].<br><br>Lieberman, M.D., *Social: Why Our Brains Are Wired To Connect*. 2014, New York, NY: Crown Publishing Group. 384 [40].
Sociobiology or Ethology	Textbook & Articles	Wilson, E., *Sociobiology: The New Synthesis*. 25th ed. 2000, Cambridge, MA and London, England: The Belknap Press of Harvard University Press. 910 [41].<br><br>Tinbergen, N., *On aims and methods of Ethology.* Animal Biology, 2005. 55(4): p. 297-321 [42].<br><br>Strassmann, J.E., *Tribute to Tinbergen: The Place of Animal Behavior in Biology.* Ethology, 2014. 120(2): p. 123-126 [43].<br><br>Farina, S. and M. Gibbons, *The Last Refuge of Scoundrels: New Evidence of E.O. Wilson's Intimacy with Scientific Racism*, in *Science for the People*. 2022 [44].

References

1. U.S. Department of Health and Human Services. Our Epidemic of Loneliness and Isolation: The U.S. Surgeon General's Advisory on the Healing Effects of Social Connection and Community. 2023. Available online: https://www.hhs.gov/sites/default/files/surgeon-general-social-connection-advisory.pdf (accessed on 2 June 2024).
2. Modell, H.; Cliff, W.; Michael, J.; McFarland, J.; Wenderoth, M.P.; Wright, A. A physiologist's view of homeostasis. *Adv Physiol Educ*. **2015**, *39*, 259-66. https://doi.org/10.1152/advan.00107.2015.
3. Billman, G.E. Homeostasis: The Underappreciated and Far Too Often Ignored Central Organizing Principle of Physiology. *Frontiers in Physiology*. **2020**, *11*, 200. https://doi.org/10.3389/fphys.2020.00200.
4. Cacioppo, J.T.; Patrick, W. *Loneliness: Human nature and the need for social connection*; W. W. Norton & Company: New York, NY and London, UK, 2008; p. 264.
5. Coan, J.A.; Sbarra, D.A. Social Baseline Theory: The Social Regulation of Risk and Effort. *Curr Opin Psychol*. **2015**, *1*, 87-91. https://doi.org/10.1016/j.copsyc.2014.12.021.
6. Matthews, G.A.; Tye, K.M. Neural mechanisms of social homeostasis. *Ann N Y Acad Sc*. **2019**, *1457*, 5-25. https://doi.org/10.1111/nyas.14016.
7. Fulford, D.; Holt, D.J. Social Withdrawal, Loneliness, and Health in Schizophrenia: Psychological and Neural Mechanisms. *Schizophr Bull*. **2023**, *49*, 1138-1149. https://doi.org/10.1093/schbul/sbad099.
8. Bronfenbrenner, U. Toward an experimental ecology of human development. *Am Psychol*. **1977**, *32*, 513-531.
9. U.S. Centers for Disease Control and Prevention. The Social-Ecological Model: A Framework for Prevention. 2022. Available online: https://www.cdc.gov/violenceprevention/about/social-ecologicalmodel.html (accessed in 1 June 2024).
10. Gudi-Mindermann, H.; White, M.; Roczen, J.; Riedel, N.; Dreger, S.; Bolte, G. Integrating the social environment with an equity perspective into the exposome paradigm: A new conceptual framework of the Social Exposome. *Environ Res*. **2023**, *233*, 116485. https://doi.org/10.1016/j.envres.2023.116485.
11. The National Institute of Environmental Health Sciences. Exposure Science. 2023. Available online: https://www.niehs.nih.gov/health/topics/science/exposure/index.cfm (accessed on 1 June 2024).
12. Raphael, D. (Ed.) *Social Determinants of Health: Canadian Perspective*, 3rd ed. Canadian Scholars Press Inc.: Toronto, ON, Canada, 2015.
13. Raphael, D.; Bryant, T. Emerging Themes in Social Determinants of Health Theory and Research. *Int J Health Serv*. **2022**, *52*, 428-432. https://doi.org/10.1177/00207314221109515.
14. Shultz, J.M.; Sullivan, L.M.; Galea, S. *Public Health*, 2nd ed.; An Introduction to the Science and Practice of Population Health; Springer Publishing Company: New York, NY, USA, 2023. https://doi.org/10.1891/9780826180438.
15. Muntaner, C.; Benach, J. Why social (political, economic, cultural, ecological) determinants of health? Part 1: Background of a contested construct. Int. J. Soc. Determ. Health Health Serv. **2023**, *53*, 117-121. https://doi.org/10.1177/27551938231152996.

16. Centers for Disease Control and Prevention. *Why is addressing social determinants of health important for CDC and Public Health?* 2022. Available online: https://www.cdc.gov/about/priorities/why-is-addressing-sdoh-important.html?CDC_AAref_Val=https://www.cdc.gov/about/sdoh/addressing-sdoh.html (accessed on 27 February 2025).
17. World Health Organization. Social Determinants of Health. 2022. Available online: https://www.who.int/health-topics/social-determinants-of-health#tab=tab_1 (accessed on 1 June 2024).
18. Reader, S. (Ed.) A Philosophy of Need; Talking Philosophy. Cambridge University Press: Cambridge, UK, 2024. https://doi.org/10.1017/9781009230179.
19. Goldberg, D.S. Public Health Ethics and the Social Determinants of Health, 1st ed. SpringerBriefs in Public Health: Aurora, CO, USA, 2017. Available online: http://naturalisms.org/epidemic-ethics/Goldberg%202017%20Public%20Health%20Ethics%20and%20the%20Social%20Determinants%20of%20Health.pdf (accessed on 27 February 2025).
20. Holt-Lunstad, J.; Smith, T.B.; Layton, J.B. Social relationships and mortality risk: a meta-analytic review. *PLoS Med*. **2010**, *7*, e1000316. https://doi.org/10.1371/journal.pmed.1000316.
21. Holt-Lunstad, J.; Smith, T.B.; Baker, M.; Harris, T. Loneliness and social isolation as risk factors for mortality: a meta-analytic review. *Perspect Psychol Sci*. **2015**, *10*, 227-37. https://doi.org/10.1177/1745691614568352.
22. Holt-Lunstad, J. Why Social Relationships Are Important for Physical Health: A Systems Approach to Understanding and Modifying Risk and Protection. *Annu Rev Psychol*. **2018**, *69*, 437-458. https://doi.org/10.1146/annurev-psych-122216-011902.
23. Holt-Lunstad, J. Social Connection as a Public Health Issue: The Evidence and a Systemic Framework for Prioritizing the "Social" in Social Determinants of Health. *Annu Rev Public Health*. **2022**, *43*, 193-213. https://doi.org/10.1146/annurev-publhealth-052020-110732.
24. McEwen, B.S. Stress, adaptation, and disease. Allostasis and allostatic load. *Ann N Y Acad Sci*. **1998**, *840*, 33-44. https://doi.org/10.1111/j.1749-6632.1998.tb09546.x.
25. McEwen, B.S. Stressed or stressed out: what is the difference? *J Psychiatry Neurosci*. **2005**, *30*, 315-8.
26. McEwen, B.S.; Gianaros, P.J. Stress- and allostasis-induced brain plasticity. *Annu Rev Med*. **2011**, *62*, 431-45. https://doi.org/10.1146/annurev-med-052209-100430.
27. McEwen, C.A. Connecting the biology of stress, allostatic load and epigenetics to social structures and processes. *Neurobiol Stress*. 2022, *17*, 100426. https://doi.org/10.1016/j.ynstr.2022.100426.
28. Hawkley, L.C.; Browne, M.W.; Cacioppo, J.T. How Can I Connect With Thee?: Let Me Count the Ways. *Psychological Science*. **2005**, *16*, 798-804. https://doi.org/10.1111/j.1467-9280.2005.01617.x.
29. Hawkley, L.C.; Cacioppo, J.T. Loneliness matters: a theoretical and empirical review of consequences and mechanisms. *Ann Behav Med*. **2010**, *40*, 218-27. https://doi.org/10.1007/s12160-010-9210-8.
30. Cacioppo, S.; Grippo, A.J.; London, S.; Goossens, L.; Cacioppo, J.T. Loneliness: clinical import and interventions. *Perspect Psychol Sci*. **2015**, *10*, 238-49. https://doi.org/10.1177/1745691615570616.

31. Cacioppo, J.T.; Cacioppo, S. The growing problem of loneliness. *Lancet*. **2018**, *391*, 426. https://doi.org/10.1016/s0140-6736(18)30142-9.
32. Krieger, N. Embodiment: a conceptual glossary for epidemiology. *J Epidemiol Community Health*. **2005**, *59*, 350-5. https://doi.org/10.1136/jech.2004.024562.
33. Krieger, N. Discrimination and health inequities. *Int J Health Serv*. **2014**, *44*, 643-710. https://doi.org/10.2190/HS.44.4.b.
34. Krieger, N. Living and Dying at the Crossroads: Racism, Embodiment, and Why Theory Is Essential for a Public Health of Consequence. *Am J Public Health*. **2016**, *106*, 832-3. https://doi.org/10.2105/ajph.2016.303100.
35. Krieger, N. Theorizing epidemiology, the stories bodies tell, and embodied truths: a status update on contending 21(st) c CE epidemiological theories of disease distribution. *Int J Soc Determinants Health Health Serv*. **2024**, *54*, 331-342. https://doi.org/10.1177/27551938241269188.
36. Sher, A.A. *Introduction to Conservation Biology*, 3rd ed. Oxford University Press: Oxford, UK, 2022.
37. Sher, A.; Molles, M.C., Jr. *Ecology: Concepts and Applications*, 9th ed. McGraw Hill: New York, NY, USA, 2022; p. 547.
38. Willmer, P.; Stone, G.; Johnston, I. *Environmental physiology of animals*. John Wiley & Sons: Hoboken, NJ, USA, 2009.
39. *Social Epidemiology*, 2nd ed.; Berkman, L., Kawachi, I., Maria Glymour, M., Eds.; Oxford University Press: New York, NY, USA 2014.
40. Lieberman, M.D. *Social: Why Our Brains Are Wired To Connect;* Crown Publishing Group: New York, NY, USA, 2014; p. 384.
41. Wilson, E. *Sociobiology: The New Synthesis*, 25th ed.; The Belknap Press of Harvard University Press: Cambridge, MA, USA; London, UK, 2000; p. 910.
42. Tinbergen, N. On aims and methods of Ethology. *Anim. Biol.* **2005**, *55*, 297-321. https://doi.org/10.1163/157075605774840941.
43. Strassmann, J.E. Tribute to Tinbergen: The Place of Animal Behavior in Biology. *Ethology*. **2014**, *120*, 123-126. https://doi.org/10.1111/eth.12192.
44. Farina, S.; Gibbons, M. The Last Refuge of Scoundrels: New Evidence of E.O. Wilson's Intimacy with Scientific Racism. *Science for the People*. 2022. Available online: https://magazine.scienceforthepeople.org/online/the-last-refuge-of-scoundrels/ (accessed on 27 February 2025).
